# Enhancer-mediated enrichment of interacting JMJD3–DDX21 to *ENPP2* locus prevents R-loop formation and promotes transcription

**DOI:** 10.1093/nar/gkz560

**Published:** 2019-06-28

**Authors:** Deborah Argaud, Marie-Chloé Boulanger, Arnaud Chignon, Ghada Mkannez, Patrick Mathieu

**Affiliations:** Laboratory of Cardiovascular Pathobiology, Quebec Heart and Lung Institute/Research Center, Department of Surgery, Laval University, Quebec G1V-4G5, Canada

## Abstract

*ENPP2*, which encodes for the enzyme autotaxin (ATX), is overexpressed during chronic inflammatory diseases and various cancers. However, the molecular mechanism involved in the *ENPP2* transcription remains elusive. Here, in HEK 293T cells, we demonstrated that lipopolysaccharide (LPS) increased the transcription process at *ENPP2* locus through a NF-кB pathway and a reduction of H3K27me3 level, a histone repressive mark, by the demethylase UTX. Simultaneously, the H3K27me3 demethylase JMJD3/KDM6B was recruited to the transcription start site (TSS), within the gene body and controlled the expression of *ENPP2* in a non-enzymatic manner. Mass spectrometry data revealed a novel interaction for JMJD3 with DDX21, a RNA helicase that unwinds R-loops created by nascent transcript and DNA template. Upon LPS treatment, JMJD3 is necessary for DDX21 recruitment at *ENPP2* locus allowing the resolution of aberrant R-loops. CRISPR-Cas9-mediated deletion of a distant-acting enhancer decreased the expression of *ENPP2* and lowered the recruitment of JMJD3–DDX21 complex at TSS and its progression through the gene body. Taken together, these findings revealed that enhancer-mediated enrichment of novel JMJD3–DDX21 interaction at *ENPP2* locus is necessary for nascent transcript synthesis via the resolution of aberrant R-loops formation in response to inflammatory stimulus.

## INTRODUCTION

Autotaxin (ATX), which is encoded by *ENPP2* gene, is an extracellular lysophospholipase D that hydrolyzes lysophosphatidylcholine (LPC) into lysophosphatidic acid (LPA), a lipid mediator acting on specific G protein-coupled receptors (GPCRs). ATX-LPA signaling is essential for embryonic development and has been implicated in various cancer processes and chronic inflammation (1,2). However, the molecular mechanisms whereby inflammatory stimuli control the expression of *ENPP2* are poorly understood. Two DNA sequences known as κB sites (5′-GGGRNNYYCC-3′; R, purine; Y, pyrimidine; N, any nucleotide) at the *ENPP2* promoter suggests a role for the nuclear factor kappa B (NF-κB) in the regulation of *ENPP2* transcription (3). NF-κB pathway orchestrates the inflammatory response by regulating a complex transcriptional program including hundreds of genes involved in cell survival, differentiation and proliferation (4,5). Chromatin compaction, DNA accessibility for transcription factors and also the recruitment of transcription machinery are modulated by histones modifications. Multiple lysine residues within histones are subject to methylation such as trimethylation on lysine 27 of histone H3 (H3K27me3). This histone mark, associated with transcriptional repression, is deposited by EZH2, a methyl transferase part of the Polycomb Repressive Complex 2 (PRC2) (6–8). Two histone lysine demethylases, including ubiquitously transcribed tetratricopeptide protein, X-linked (UTX) and jumonji domain-containing 3 (JMJD3) are known to govern the removal of H3K27me3 (9,10).

Previous studies revealed that JMJD3 expression is quickly induced by NF-κB in primary mouse macrophages in response to lipopolysaccharide (LPS) and fine-tunes the transcriptional gene patterns in an H3K27 demethylation-independent manner (11). In other cellular context, JMJD3 can regulate the transcription elongation by promoting the release of paused RNA Polymerase II (RNA pol II), and its progression along gene body (12,13).

During the transcription elongation step, nascent RNA can reinvade the DNA double helix, and hybridize with the DNA template, resulting in a DNA–RNA hybrid called R-loops, which is accompanied by a single-stranded DNA (ssDNA) (14,15). Genome-wide profiling approaches revealed that R-loops are highly abundant: 5% of the human genome has the potential to form R-loops (16). These structures are enriched in promoter, gene bodies and in terminator regions (15,17–19). Interestingly, the functions of R-loops seem dependent on their genomic context. For instance, R-loops at promoter region ensure transcription factor binding or block DNA methylation, whereas it was speculated that R-loops over exon 2 would favor Pol II pausing (17,20,21).

As excessive R-loops formation represents an obstacle for transcription and leads to DNA damage, human cells possess mechanisms that tightly control R-loop levels (22). RNA processing factors and members of RNase H family that specifically degrade the RNA in R-loops are essential for genome stability. Another mechanism to prevent R-loop formation is the activity of RNA–DNA helicases which efficiently unwind R-loops using the energy from adenosine triphosphate hydrolysis. Interestingly, inactivation of helicase senataxin (SETX) leads to increased occurrences of R loops at transcription termination pause sites and increased DNA damage (23). Growing evidence point to an important role of the DEAD (Asp–Glu–Ala–Asp)-box family of RNA helicases in R-loops biology, as seen in diverse cellular contexts. Upon DNA damage, DDX19 transiently relocalizes to the nucleus to resolve R‐loops arising from conflicts between transcription and replication (24). Alternatively, during transcription elongation, DDX23 acts to release RNA Pol II from R-loop-mediated pausing throughout the gene body (25). Also, depletion of DDX21 leads to aberrant R loops formation, stalling of RNA polymerases and accumulation of γH2AX foci (26).

In this work, we show that *ENPP2* is a bivalent gene, marked by H3K4me3 and H3K27me3 and is associated with promoter-proximal stalling of RNA pol II before activation. During inflammation, UTX promotes the removal of H3K27me3 at the promoter, whereas JMJD3 is recruited as part of a complex with DDX21 at transcription start site (TSS) and controls the expression of *ENPP2* in a nonenzymatic manner. Mechanistically, enrichment of novel JMJD3–DDX21 interaction at *ENPP2* locus is promoted by a DNA loop between a distant-acting enhancer and *ENPP2* promoter allowing the resolution of aberrant R-loops, a process necessary for nascent transcript synthesis at *ENPP2* locus.

## MATERIALS AND METHODS

### Real-time polymerase chain reaction

RNA was extracted from HEK 293T cells and isolated with E.Z.N.A Total RNA Kit I from Omega (VWR, Canada). The RNA extraction protocol was performed according to manufacturer's instructions. One microgram of RNA was reverse transcribed using the Qscript cDNA supermix from Quanta (VWR, Canada). Quantitative real-time polymerase chain reaction (qPCR) was performed with perfecta sybr supermix from Quanta on the Rotor-Gene 6000 system (Corbett Robotics Inc, CA, USA). Primers for *ENPP2* (cat no.QT00044387) were obtained from QIAGEN (ON, Canada). The expression of the hypoxanthine-guanine phosphoribosyltransferase (HPRT, cat no. QT00059066) gene (QIAGEN, ON, Canada) was used as a reference to normalize the results.

### Cell transfection with siRNA and plasmids

HEK 293T cells were seeded on poly-l-lysine (Sigma-Aldrich, Oakville, ON, Canada) and transfected with plasmid using the calcium phosphate technique. The vector pGL033-p65 was a kind gift from Dr Warner Greene lab (Gladstone Institute of Virology and Immunology, San Francisco, USA). For JMJD3 rescue experiments plasmids encoding for JMJD3 wild-type and mutant JMJD3 H1390A were kindly shared with us by Dr Charlie Degui Chen lab (Institute of Biochemistry and Cell Biology, Shangaï, China) (27). For DDX21, wild-type plasmid was ordered from GeneCopoeia (cat# EX-E1618-M08, MD, USA). Mutant DDX21 S375L/A376E (28,29) was created by site-directed mutagenesis (Civic Bioscience Ltee, QC, Canada). The vector RnaseH (#108699) and the plasmid encoding for HA-JMJD3 (#24167) used for analysis of the interactome of JMJD3 were obtained from Addgene (MA, USA). For siRNA treatment, cells were transfected by use of Lipofectamine RNAiMax (Invitrogen Thermofisher, Canada). siRNAs targeting JMJD3 (siJMJD3#1 cat no. #SI03181136 and siJMJ3#2 cat no.#SI04133836), DDX21 (siDDX21#1 cat no. #SI04311412 and siDDX21#2 cat no.#SI04155781), UTX (siUTX#1 cat no#SI04306743 and siUTX#2 cat no. #SI04314303), and EZH2 (cat no. #SI02665166) were purchased from QIAGEN (ON, Canada). For rescue experiments, HEK293T cells were transfected with siRNA targeting the 3′untranslated region (UTR) of intended mRNA target, followed by expression of siRNA-resistant wild-type or mutants constructs for DDX21 and JMJD3.

### Luciferase reporter assay

HEK 293T cells were transfected with vectors encoding for 2.5 kb of ENPP2 promoter. In some constructions κB1 (CTGGCATTTCCAGTAT) or/and κB2 (CTGGTGGAAAGCCCTT) sites were deleted by directed mutagenesis. For analysis of enhancer activity, HEK 293T cells were transfected with the pGL4.10 luciferase vector (Promega, WI, USA) containing a minimal promoter sequence with or without the enhancer region located at chr8:120733232-120735226 on build 37 from ENCODE (Gene synthesis and subcloning, Bio Basic, ON, Canada). In these experiments, a vector coding for renilla luciferase was used as a reporter for transfection efficiency. Luciferase activities were measured at 48 h after transfection with the Promega Dual-Luciferase Reporter Assay System (Promega, Fitchburg, WI, USA) according to the manufacturer's protocol.

### Chromatin immunoprecipitation (ChIP) and detection of R-loops

HEK 293T cells were crosslinked by adding formaldehyde solution to a final concentration 0.2%, dropwise directly to the media for 10 min at room temperature (Sigma-Aldrich). Then cells were harvest in 1 ml of phosphate-buffered saline (PBS) 1× (Sigma, ON, Canada) and were centrifuged 5 min at 800 *g* at 4°C. The supernatants were removed, pellets were then resuspended in 500 μl lysis buffer (50 mM Hepes-KOH, pH7.5, 140 mM NaCl, 1 mM ethylenediaminetetraacetic acid (EDTA) pH8.0, 1% triton X-100, 0.1% sodium deoxycholate, 0.1% sodium dodecyl sulphate (SDS), PIC) and sonicated to shear the DNA to an average length of 100–400 base pairs (bps). One microgram of H3K27me3 (#9733, Cell signaling), 2 μg of H3K4me3 (#9751, Cell signaling), 2 μg of H3K4me1 (#5326, Cell signaling), 8 μg of RNA pol II (#Ab26721, Abcam), 8 μg of UTX (#Ab36938, Abcam), 8 μg of JMJD3 (#Ab38113, Abcam), 2 μg p65 (#3012-100, Biovision), 2 μg of DDX21 (#NBP1-83310, Novus Biologicals) antibodies or isotype IgG (Cell Signaling, MA, USA) were incubated with proteins G dynabeads (Life technologies, Thermofisher, ON, Canada) for 6 h before DNA samples were added to the antibodies/dynabeads mixtures and incubated at 4°C overnight on a rotator. R-loops detection was performed using 8 μg of S9.6 (#ENH001, Kerafast) antibody as previously described (30–34). The next day, the samples were washed with low salt buffer (0.1% SDS, 1% triton X100, 2 mM EDTA, 20 mM Tris–HCl pH 8.0 and 150 mM NaCl), then with high salt buffer (0.1% SDS, 1% triton X100, 2 mM EDTA, 20 mM Tris–HCl pH 8.0 and 500 mM NaCl), followed with LiCl buffer (10 mM Tris–HCl, pH8.0, 250 mM LiCl, 1 mM EDTA, pH 8.0, 1% NP-40) and finally with TE buffer (10 mM Tris–HCl, pH8.1, 1 mM EDTA, pH 8.0). The DNA–proteins complex were then eluted from dynabeads by adding 200 μl of elution buffer (1% SDS, 100 mM NaHCO_3_) at 65°C for 1 h. Eluted samples were reverse cross-linked at 65°C overnight by adding 4.8 μl of 5M NaCl. The final mixture containing DNA was digested with 0.33 mg/ml of RNase A (QIAGEN, ON, Canada) for 1 h at 37°C and proteins digested for 1 h at 55°C with 0.5 mg/ml of proteinase K (QIAGEN, ON, Canada). Samples were extracted by phenol–chloroform and analyzed by quantitative PCR assay using primers listed in Supplementary Table S1.

### ChIP-ReChIP

Re-chromatin immunoprecipitation (Re-ChIP) assays was performed according the same protocol except one step. Briefly, HEK 293T cells were crosslinked, lysed and sonicated as described in the ChIP protocol. Chromatin was incubated with first antibody (anti-HA) in the presence of magnetic beads. The beads were washed, and the material was eluted with Re-CHIP elution buffer (2 mM EDTA, 500 mM NaCl, 0.1% SDS, 1% NP-40) at 37°C for 30 min. Eluted samples were further incubated with the second antibody (anti-DDX21 or IgG).

### Co-immunoprecipitation

HEK 293T were transfected with HA-JMD3 plasmids and harvested with TNGT buffer (150 mM NaCl, 50 mM Tris–HCl pH 7.4 1M, 1% Glycerol, 0.1% Triton X100, PIC). Whole cellular extracts were treated with 40U/ml of RNaseH, 25U/ml of DNaseI for 1 h at 37°C or not and immunoprecipitated with 8 μg of antibodies against HA-epitope (Sigma-Aldrich, ON, Canada), or isotype IgG (Cell Signaling, MA, USA) in the presence of 40μl magnetic beads (BioRad, CA, USA) overnight at 4°C. The next day, samples were washed three times with TNGT. Purified proteins were analyzed by western blot.

### Western blot

Samples were boiled for 5 min, and proteins were loaded onto polyacrylamide gels followed by electrophoresis and transferred onto nitrocellulose membranes. Membranes were blocked with TBS-tween containing 5% non-fat dry milk and incubated with either HA primary antibodies (#H9658, Sigma-Aldrich), RNA polymerase II (#Ab26721, Abcam), DDX21 (#NBP1-83310, Novus Biologicals) using a 1:1000 dilution, overnight at 4°C. Membranes were then washed and incubated with HRP-labeled secondary antibodies (Cell Signaling Technology, MA, USA) using a 1:2000 dilution. Detection was done using clarity western ECL substrate (BioRad, ON, Canada).

### Purification and identification of JMJD3-associated proteins

Fifty million cells were used per condition and were transfected with a plasmid coding for HA-JMJD3 for 24 h. LPS treatment was performed for 6 h at 100 ng/ml. The cells were then pelleted at 1200 *g* for 5 min (4°C) and the supernatant removed. The resulting pellet was then resuspended in 10 ml of TNGT buffer (150 mM NaCl, 50 mM Tris–HCl pH 7.4 1M, 1% Glycerol, 0.1% Triton X100, PIC) and immunoprecipitated with 360 μg of antibodies against HA-epitope (Sigma-Aldrich, ON, Canada) conjugated to 1800 μl of magnetic beads (BioRad, CA, USA). This IP mixture was then incubated overnight at 4°C (rotating). The next day, samples were washed three times with TNGT, and three times with ammonium bicarbonate 50 mM (freshly prepared). After the last wash, the beads were briefly spun down and any residual liquid was removed. Beads were frozen (−20°C) and later processed for mass spectrometry analysis (Proteomics platform, CHU de Québec Research Center, Qc, Canada). The MS/MS spectra, were filtered by the ‘Exclusive Unique Peptide Count’ from Scaffold3 software, corresponding to the number of different amino acid sequences (unique peptides), regardless of any modification that are attributed to a single protein. Raw data are provided in Supplementary Table S2. Peptides-associated with JMJD3 were considered valid if they contained more than two unique peptide fragments and matches of 95% probability. At last, data were processed with a Gene Ontology (GO) analysis from David interface (https://david.ncifcrf.gov/summary.jsp).

### Capture nascent RNAs

To capture nascent RNAs, HEK 293T cells were labeled by adding 0.2 mM ethynyl uridine (EU) into the culture medium for 6 h simultaneously with LPS. The EU-labeled RNA were extracted and 8 μg biotinylated with 0.8 mM biotin azide by using the Click-it Nascent RNA Capture Kit (Life technologies, Thermofisher, ON, Canada). The biotinylated RNAs were then precipitated with ethanol and 1 μg bound to 50 μl of Dynabeads MyOne Streptavidin T1 magnetic beads in Click-iT RNA binding buffer containing 80U of RNAseOUT Recombinant Ribonuclease Inhibitors (Life technologies, Thermofisher, ON, Canada). The beads were washed with Click-iT wash buffer1 and 2, resuspended in 50 μl of Click-iT wash buffer 2 and used for cDNA synthesis using SuperScript VILO cDNA synthesis kit (Life technologies, Thermofisher, ON, Canada) in accordance with the manufacturer's instructions.

### Promoter capture Hi-C (PCHi-C)

Data on *ENPP2* chromosome interactions were retrieved from the Promoter Capture HiC (www.chicp.org) (35). PCHi-C studies provided high-resolution analysis of interactions between 31 253 annotated promoters (Fantom5, ENCODE) and distal DNA regulatory elements in 17 human primary blood cell types. Data from CD34+ cells were retrieved and analyzed. The data visualization results in a circularized overview of interactions and annotations in chromosome. Each interaction is indicated with colored arcs.

### CRISPR/Cas9 editing

HEK 293T cells were transfected with Cas9-PuroR constructs #62988 (Addgene, MA, USA) along with a plasmid encoding for two RNA guide specific of enhancer region located at chr8:120733232-120735226 on build 37. After 24 h, the cells were selected with 2 μg/ml puromycin for 48 h (Wisent, QC, Canada), and q-PCR or ChIP experiments were performed. To monitor the enhancer deletion, we performed a PCR-based strategy as described previously (36,37). Briefly, we used q-PCR with five sets of primers covering the enhancer region and two additional primer sets located upstream and downstream of the predicted cut sites (control) as depicted in Supplementary Figure S9A.

### Chromosome conformation capture (3C)

Chromosome conformation capture (3C) experiments was performed as described previously (38). Briefly, 1 × 10^7^ HEK 293T cells were harvested in 10 ml of Dulbecco's-modified Eagle's medium, centrifuged 5 min at 800 *g* at 4°C and supernatants were removed. Cell pellets were resuspended in 10 ml of PBS 1×/SVF 10% and cross-linked by adding paraformaldehyde solution to a final concentration 1%, directly to the media for 15 min at room temperature (Sigma-Aldrich). Quenching step was performed by adding 1.425 ml of 1M glycine. Next, cells were centrifuged 5 min at 400 *g* at 4°C, resuspended in 5 ml of lysis buffer (10 mM Tris–HCl pH7.5; 10mM NaCl; 0.2% NP40; PIC 1×) and incubated for 10 min on ice. Nuclei were isolated by centrifugation for 5 min at 400 *g*, resuspended with 500 μl of CutSmart Buffer 1.2× (New England BioLabs) supplemented with 0.3% SDS. Nuclei were incubated 1 h at 37°C while being shaken at 900 r.p.m. before the addition of 50 μl of 20%Triton X-100 (2% final) and an additional incubation was performed. DNA was then digested overnight at 37°C with 400U of NspI (New England BioLabs, ON, Canada). The next day, restriction enzyme was inactivated by the addition of 1.6% SDS at 65°C for 25 min and digested nuclei were diluted 20-fold with T4 DNA Ligase buffer 1.15× (New England BioLabs, ON, Canada) containing 1% of Triton X-100. Ligation step was performed with 400U of T4 DNA Ligase (New England BioLabs, ON, Canada) at 16°C overnight. Samples were reverse cross-linked at 65°C overnight in presence of proteinase K. The final mixture containing DNA was digested with 300 μg of RNaseA (QIagen, ON, canada), extracted by phenol-chloroform. Quantification of ligated products was performed by qPCR using the NspI cutting site, located in the TSS of *ENPP2* as an anchor primer combined with primers positioned to each NspI cutting site. The sequences of primers used are listed in Supplementary Table S1. Results were expressed as frequency of random interactions between sites separated by increasing genomic distances ranging from 0.3 to 85 kb with the anchor primer. For all fragments, we first normalized using a *GAPDH* loading control, as described previously (38). Second, we used the level of ligation products between the anchor and the nearest downstream NspI site (#1) as a strong normalizer to quantify interaction frequency. qPCRs were performed in quadruplicates to improve sensitivity. Enhancer-promoter interactions are demonstrated as a peak across a region of multiple primers

### Statistical analysis

Continuous data were expressed as mean ± SEM and compared by use of the Student *t*-test (when two groups were compared) or 1-way ANOVA to test the effect of group (when >2 groups were compared). A value of *P* < 0.05 was considered significant. Statistical analysis was performed with a commercially available software package JMP 10.0 or Prism 6.0.

## RESULTS

### NF-κB response elements mediate the expression of *ENPP2*

**Figure 1.**
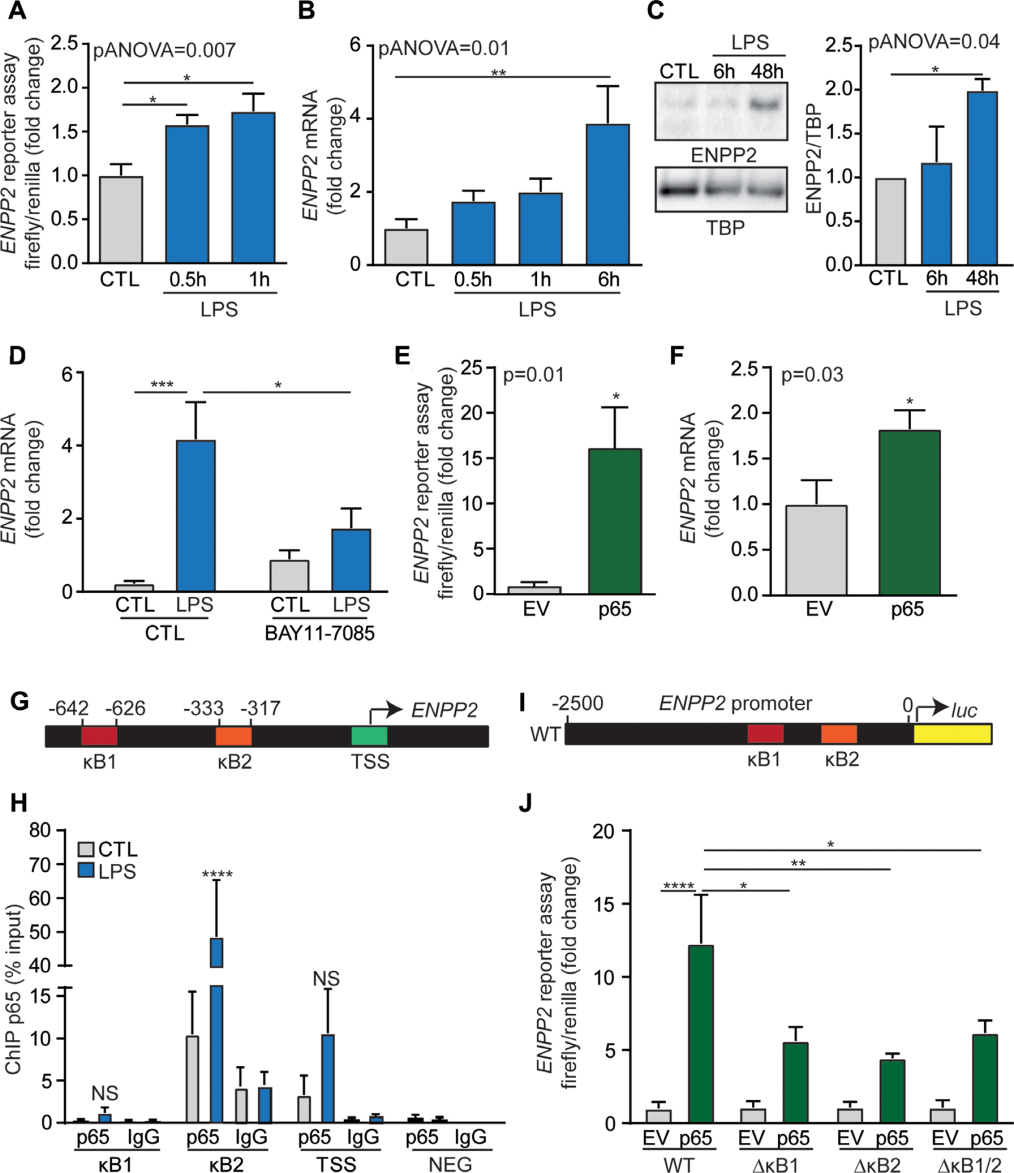
NF-κB response elements mediate the expression of *ENPP2*. (**A**) Luciferase assay demonstrating increased transcriptional activity at the *ENPP2* promoter following LPS treatment for the indicated time. (**B** and **C**) Endogenous *ENPP2* expression in HEK 293T cells treated with LPS at the indicated time points (B) qPCR analysis (C) representative western blots and quantifications. (**D**) Effect of a 24 h treatment with BAY11-7085 on LPS-mediated *ENPP2* expression, qPCR analysis. (**E**) Effect of p65 overexpression on *ENPP2* promoter activity as shown by luciferase reporter assay. (**F**) Expression of endogenous *ENPP2* following p65 overexpression in HEK 293T, qPCR analysis. (**G**) Scheme of the two кB consensus sites localization in the *ENPP2* promoter. (**H**) ChIP-qPCR measurement of p65 enrichment at the *ENPP2* locus in HEK 293T cells treated with LPS for 6 h. A set of primer targeting an intergenic region downstream of *SNRPN* was used a negative control (NEG). (**I**) Scheme of the *ENPP2* luciferase reporter. (**J**) Luciferase reporter assay showing the effect of the кB sites deletion on *ENPP2* promoter activity. LPS 100ng/ml, BAY11-7085 1 μM.

HEK 293T cells were transfected with a luciferase reporter containing 2.5 kb of the promoter region of *ENPP2*. In time series experiments, we found that the luciferase activity was increased at 30 and 60 min after LPS treatment (Figure 1A). *ENPP2* mRNA level was also significantly increased at 6 h after the addition of LPS to the culture medium and translated into a significant rise of protein level (Figure 1B and C). LPS-mediated expression of *ENPP2* was negated by BAY11-7085, an inhibitor of NF-κB pathway (Figure 1D). Also, the overexpression of p65 (Supplementary Figure S1A and B), a NF-κB family member, robustly increased by 15-fold the reporter activity and 1.8-fold the expression of *ENPP2* (Figure 1E and F). Analysis of the promoter region of *ENPP2* revealed the presence of two NF-κB consensus sequences located at −642/−626 (кB 1) and at −333/−317 (кB 2) (Figure 1G) (3). Following a treatment with LPS, we observed by using quantitative chromatin immunoprecipitation (ChIP-qPCR) assay an enrichment of p65 at κB consensus sequences in the *ENPP2* promoter and in one site near the TSS (Figure 1H). In these experiments, a set of primers targeting an intergenic region downstream of *SNRPN* was used as negative control region. (Figure 1H).

Next, to investigate the role of these κB consensus sequences in the regulation of *ENPP2*, we deleted them in the luciferase construction containing 2.5 kb of ENPP2 promoter region (Figure 1I). In these experiments, p65-mediated activation of 2.5kb *ENPP2* promoter was largely abrogated by the deletion of κB sites, compared to *ENPP2* promoter wild-type (Figure 1J). The deletion of one κB site was sufficient to reduce the response of p65 and the effect of a double deletion was not additive. Taken together, these findings show that LPS induced the expression of *ENPP2* in HEK 293T cells through a NF-кB pathway involving the recruitment of p65 on кB consensus sequences located in the promoter region.

### *ENPP2* promoter has bivalent histone marks

**Figure 2.**
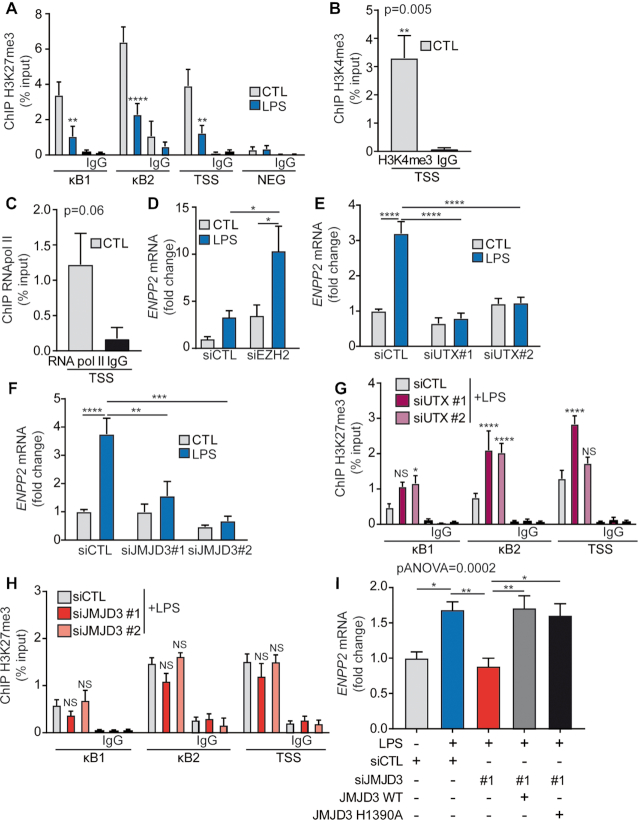
*ENPP2* promoter has bivalent histone marks. (**A**) ChIP-qPCR measurement of H3K27me3 enrichment at the *ENPP2* locus and a negative locus (NEG; downstream of *SNRPN*) in HEK 293T cells following 6 h of LPS exposure (**B** and **C**) ChIP-qPCR assays showing (B) enrichment of the H3K4me3 histone mark and (C) RNA pol II recruitment at the *ENPP2* TSS. (**D**–**F**) Effect of siRNA against (D) EZH2, (E) UTX and (F) JMJD3 on LPS mediated expression of *ENPP2*, qPCR analyses. (**G** and **H**) H3K27me3 enrichment at the *ENPP2* promoter, determined by ChIP-qPCR, in response to siRNA targeting (G) UTX and (H) JMJD3. (**I**) qPCR measurements of *ENPP2* expression in response to JMJD3 siRNA transfection followed by rescue with constructs encoding for JMJD3 wild-type or JMJD3 H1390A (catalytic dead mutant). LPS 100 ng/ml.

To investigate the chromatin changes of *ENPP2* locus associated with transcription process, we analyzed the histone modifications at *ENPP2* promoter. In ChIP-qPCR experiments, we found that the promoter region was enriched for H3K27me3 and H3K4me3 marks and bound by promoter-proximal paused RNA Pol II at the TSS (Figure 2A–C). Hence, these data suggest that *ENPP2* has characteristics of a bivalent gene: paused RNA Pol II around TSS and the coexistence of H3K27me3 with the activating histone modification H3K4me3. Interestingly, after LPS activation the level of H3K27me3 was substantially reduced at κB consensus sites and TSS (Figure 2A). We next investigated the involvement of H3K27me3-specific enzymes in the regulation of *ENPP2*: EZH2 (methyltransferase), JMJD3 (demethylase) and UTX (demethylase). The knockdown of EZH2 with small interfering RNA (siRNA) increased by 3-fold the expression of *ENPP2* after LPS stimulation (Figure 2D). Inversely, the knockdown of specific H3K27 demethylase JMJD3 and UTX using two non-overlapping siRNA prevented LPS-mediated expression of *ENPP2* (Figure 2E and F). As confirmed by q-RT-PCR and western blot, the transfection of HEK 293T cells with RNAis against EZH2, JMJD3 and UTX efficiently depleted their respective target RNA transcripts and protein levels (Supplementary Figures S2A–C, 3A–C and 4A–C). To assess whether JMJD3 and/or UTX promoted the loss of H3K27me3 at *ENPP2* locus during LPS stimulation we performed ChIP-qPCR assay with anti-H3K27me3 antibody, after a depletion of JMJD3 or UTX. The level of H3K27me3 was robustly increased with UTX depletion (Figure 2G), but not significantly different in absence of JMJD3 (Figure 2H).

To reinforce this finding, we carried out rescue experiments with JMJD3 catalytic dead mutant. First we performed a siRNA-mediated JMJD3 knockdown, and after 24 h we transfected cells again, either with a vector encoding for wild-type JMJD3, or for a catalytically inactive JMJD3 mutant (JMJD3 H1390A) (Figure 2I). Indeed, enzymatically inactive JMJD3 (JMJD3 H1390A) completely re-established the response to LPS on the expression of *ENPP2* (Figure 2I). In these experiments, we confirmed that JMJD3 mutant was similarly expressed compared to wild-type plasmid construct (Supplementary Figure S5A–C). These data are thus consistent and shows that UTX promotes the removal of H3K27me3 at the *ENPP2* locus, whereas JMJD3 controls the expression of *ENPP2* through a nonenzymatic process. At last, we investigated whether a crosstalk scenario between UTX and JMJD3 could explain these findings. Hence, we explored whether the binding of UTX to *ENPP2* promoter upon LPS treatment (Supplementary Figure S6A) was JMJD3-dependant and *vice versa* in ChIP-qPCR assays. Interestingly, gene knockdown of JMJD3 and UTX, lowered respectively the recruitment of UTX and JMJD3 at the promoter and TSS of *ENPP2* (Supplementary Figure S6B and C). These data thus indicate a co-dependency in the recruitment of JMJD3 and UTX at this locus.

### JMJD3 and DDX21 are recruited to the promoter and gene body of *ENPP2*

**Figure 3.**
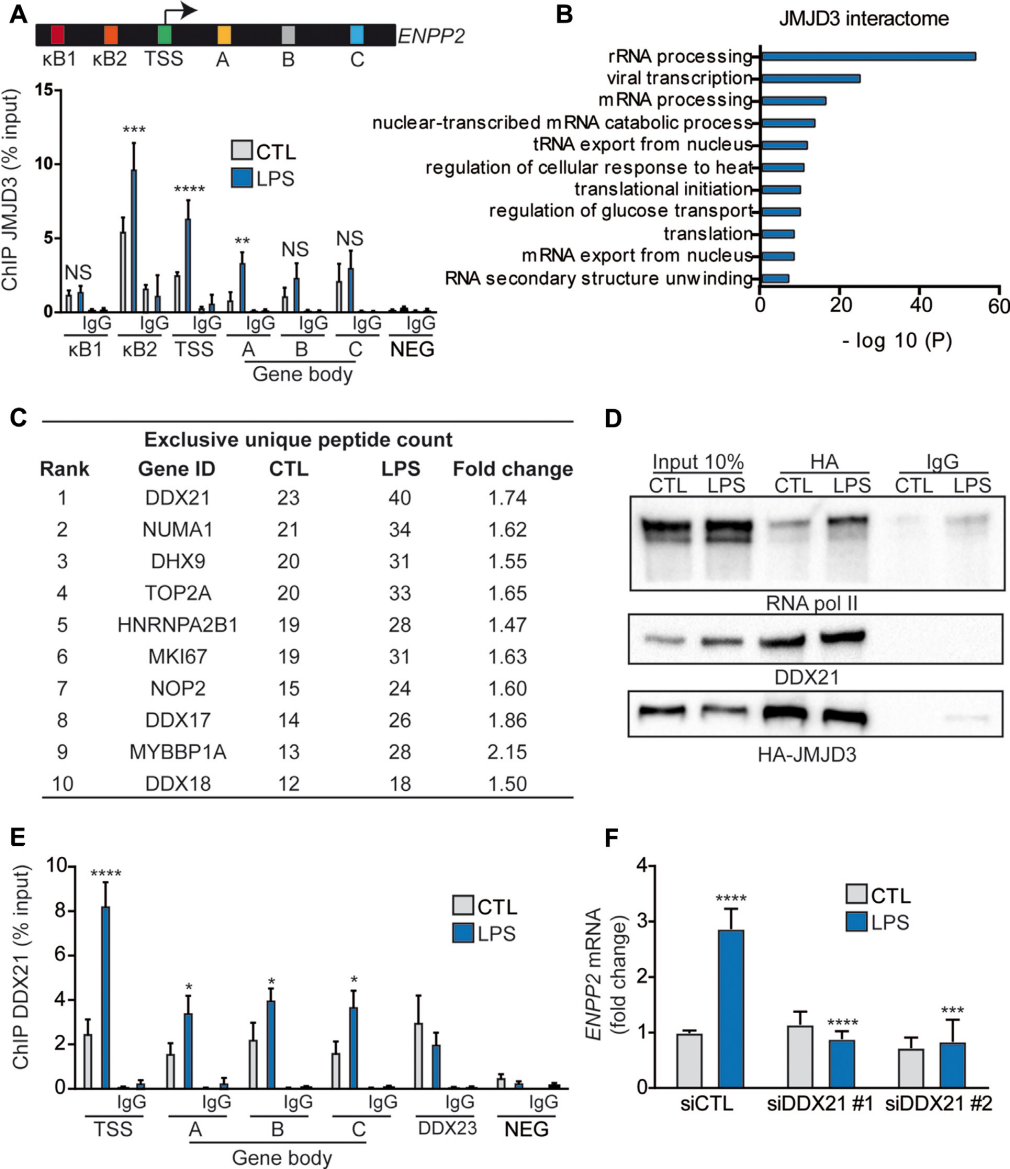
JMJD3 and DDX21 are recruited to the promoter and gene body of *ENPP2*. (**A**) JMJD3 enrichment on promoter and gene body of *ENPP2* in HEK 293T cells treated with LPS during 6 h compared to a negative locus (NEG; downstream of *SNRPN*), ChIP-qPCR analysis. (**B**) GO analysis of JMJD3 interactome identified by mass spectrometry analysis (LCMS/MS). (**C**) Table of the first ten JMJD3-associated proteins that were enriched after LPS stimulation, as determined by mass spectrometry analysis. (**D**) DDX21 and RNA pol II co-immunoprecipitated with HA-JMJD3 in HEK 293T cell extracts. (**E**) ChIP-qPCR experiment demonstrating DDX21 enrichment at *ENPP2* promoter and gene body following 6 h of LPS treatment. Two set of primers targeting an intergenic region downstream of *SNRPN (NEG;* downstream of *SNRPN)* and promoter region of DDX23 (DDX23) were used as negative and positive control, respectively. (**F**) qPCR quantification of *ENPP2* expression in response to DDX21 siRNA transfection and LPS exposure in HEK 293T cells. LPS 100 ng/ml.

Previous reports showed that JMJD3 binds to promoters, but also to gene bodies, in neural stem cells and macrophages to instruct gene expression changes by promoting RNA pol II progression (11,17). In accordance with previous data, in ChIP-qPCR, we found that in response to LPS JMJD3 was recruited to the TSS and at intron–exon junctions throughout the gene body (Figure 3A). We thus hypothesized that JMJD3 controlled the expression of *ENPP2* through its protein interactome, which could help promote transcription elongation. We transfected HEK 293T cells with a vector encoding for HA-epitope tagged JMJD3 in basal condition and after LPS stimulation and purified interaction partners by HA pull-down. Mass spectrometry results showed that 563 proteins interacted with JMJD3, and 260 were enriched after LPS treatment (with fold change ≥1.5) (Supplementary Figure S7A). Peptide coverage of specific associated proteins is shown in (Supplementary Figure S7B). A full list of JMJD3-interacting proteins is provided in Supplementary Table S2. GO analysis of JMJD3-associated proteins revealed strong association with RNA processing and unwinding of RNA secondary structure (Figure 3B). DDX21, a RNA helicase, was among the first 10 JMJD3-associated proteins that were enriched after LPS stimulation (Figure 3C). To confirm the mass spectrometry results, we performed co-immunoprecipitation experiments. Pull-down of HA-JMJD3 recovered DDX21, and an association already described with RNA Pol II (Figure 3D) (17). Interestingly, DNaseI and RNAseH treatment did not abolish the interaction between JMJD3 and DDX21 (Supplementary Figure S7C).

Previous studies have highlighted that DDX21 controls the expression of ribosomal RNA (rRNA) genes and genes transcribed by RNA pol II (26). We examined whether DDX21 could also exert a control over the expression of *ENPP2*. In ChIP-qPCR, we documented that DDX21 was recruited to the TSS and the gene body of *ENPP2* following a treatment of cells with LPS compared to negative control region (Figure 3E). In these experiments, the promoter of *DDX23*, which is bound to DDX21 was used as a positive control region (Figure 3E). In cells with siRNA-mediated knockdown of DDX21, we found that LPS-mediated expression of *ENPP2* was abrogated (Figure 3F). The efficiency of these two non-overlapping DDX21 siRNAs was confirmed by q-RT-PCR and western blot analysis (Supplementary Figure S8A–C). Hence, these data showed that JMJD3 interacts with the RNA helicase DDX21, which controls the expression of *ENPP2*.

### The complex formed by JMJD3 and DDX21 is required for R-loops resolution at *ENPP2* locus

**Figure 4.**
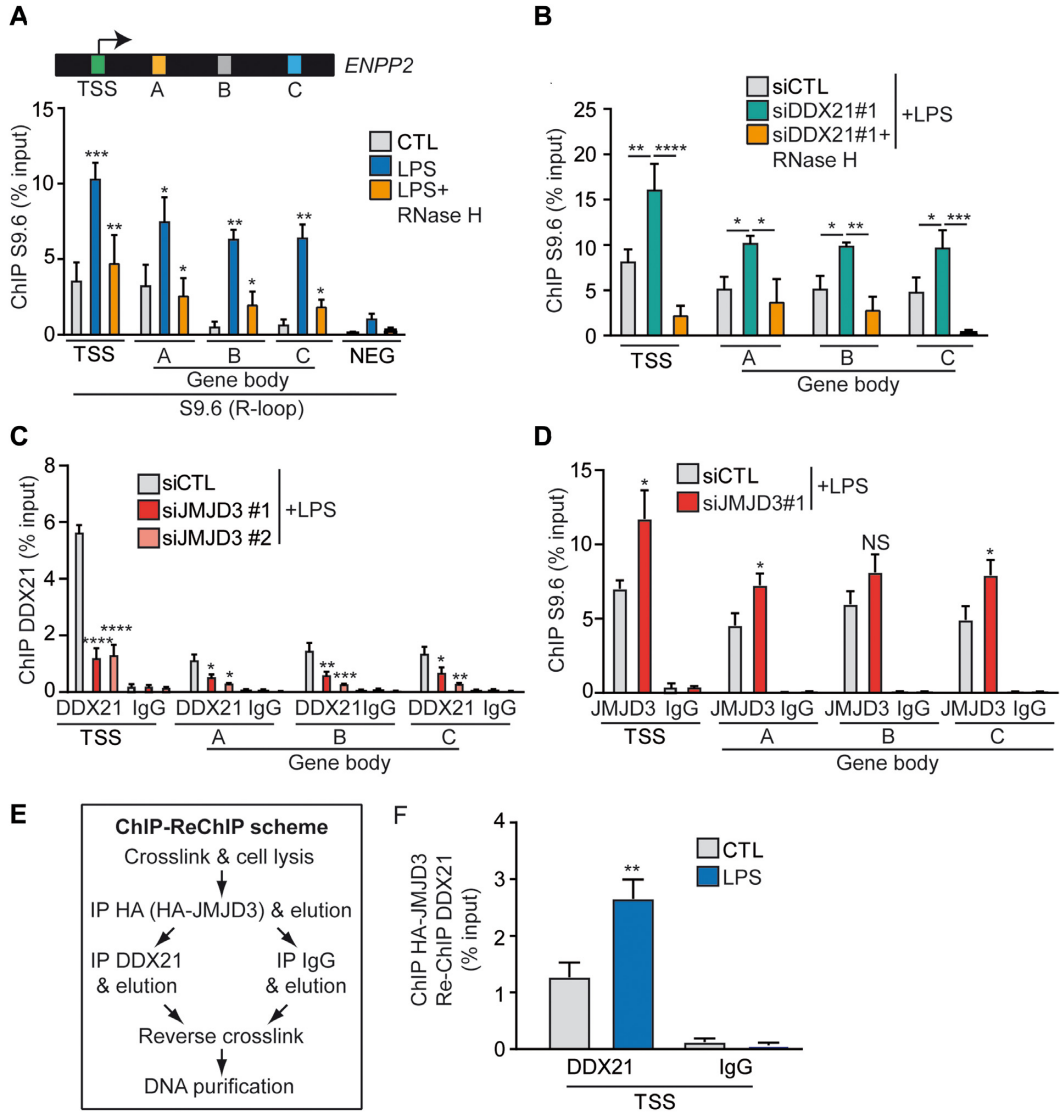
The complex formed by JMJD3 and DDX21 is required for R-loops resolution at *ENPP2* locus. (**A**) ChIP-qPCR indicating increased R-loops formation at *ENPP2* TSS, gene body and a negative locus (NEG; downstream of *SNRPN*) following LPS treatment (6 h) in HEK 293T cells; treatment of lysates with RNase H before immunoprecipitation abolished R-loops. (**B**) Effect of DDX21 siRNAs on R-loop formation, as measured by ChIP-qPCR, in presence of LPS. (**C**) JMJD3 siRNAs treatment hinders on DDX21 recruitment at *ENPP2* TSS and gene body in presence of LPS, ChIP-qPCR analysis. (**D**) ChIP-qPCR showing the effect of JMJD3 siRNA on R-loop formation at *ENPP2* TSS and gene body in the presence of LPS. (**E**) Scheme of the ChIP-ReChIP main steps. (**F**) ChIP-ReChIP-qPCR analysis indicating simultaneous binding of DDX21 and HA-JMJD3 at *ENPP2* TSS, enhanced upon addition of LPS. LPS 100 ng/ml, RNaseH 60U/ml.

Recent work has highlighted that DDX21 unwinds R-loop, thus safeguarding genome integrity and prevent stalling of RNA Pol II on estrogen-responsive genes in breast cancer cells (26). To document the presence of R-loops at the *ENPP2* locus we immunoprecipitated RNA:DNA hybrids with the S9.6 antibody (39). Interestingly, R-loops were increased after LPS treatment at TSS and within the gene body (ranged from ∼0.6 to 10% of input depending on the gene section while negative locus in intergenic region ranged from 0.1 to 1%) (Figure 4A). As expected, treatment of lysates with RnaseH before immunoprecipitation abolished the signal with S9.6 antibody and confirmed the presence of R-loops during the transcriptional process at *ENPP2* locus (Figure 4A). We next hypothesized that JMJD3 may participate to the recruitment of DDX21 at *ENPP2* locus and thus could prevent R-loop formation. Consistently, siRNA mediated knockdown of DDX21 increased R-loops formation-sensitives to RNAseH after a treatment of cells with LPS (Figure 4B). In addition, knockdown of JMJD3 led to a striking reduction in DDX21 occupancy at the TSS and gene body of *ENPP2* (Figure 4C). In these experiments, the knockdown of JMJD3 also led to a rise in R-loop formation at the TSS and gene body (Figure 4D).

To further assess whether JMJD3 and DDX21 are co-recruited at *ENPP2*, we performed ChIP/re-ChIP experiments in HEK 293T cells transfected with HA-JMJD3 plasmid and treated with LPS for 6 h. Chromatin was sequentially immunoprecipitated with anti-HA (JMJD3) and then re-chromatin immunoprecipitated (re-ChIP) with antibodies specific to DDX21 as depicted in Figure 4E. Re-ChIP experiments confirmed simultaneous binding of DDX21 and HA-JMJD3 at the TSS of *ENPP2*, suggesting that DDX21 and HA-JMJD3 might form a protein complex recruited at similar genomic region in HEK293T cells (Figure 4F). Importantly, the co-recruitment of HA-JMJD3 and DDX21 is increased by 2.5-fold after 6 h of LPS, compared to basal conditions (Figure 4F). Hence, these results show that DDX21 and JMJD3 form a complex necessary for R-loops resolution and transcription elongation upon LPS treatment.

### R-loops resolution at *ENPP2* locus promotes the synthesis of nascent transcript

**Figure 5.**
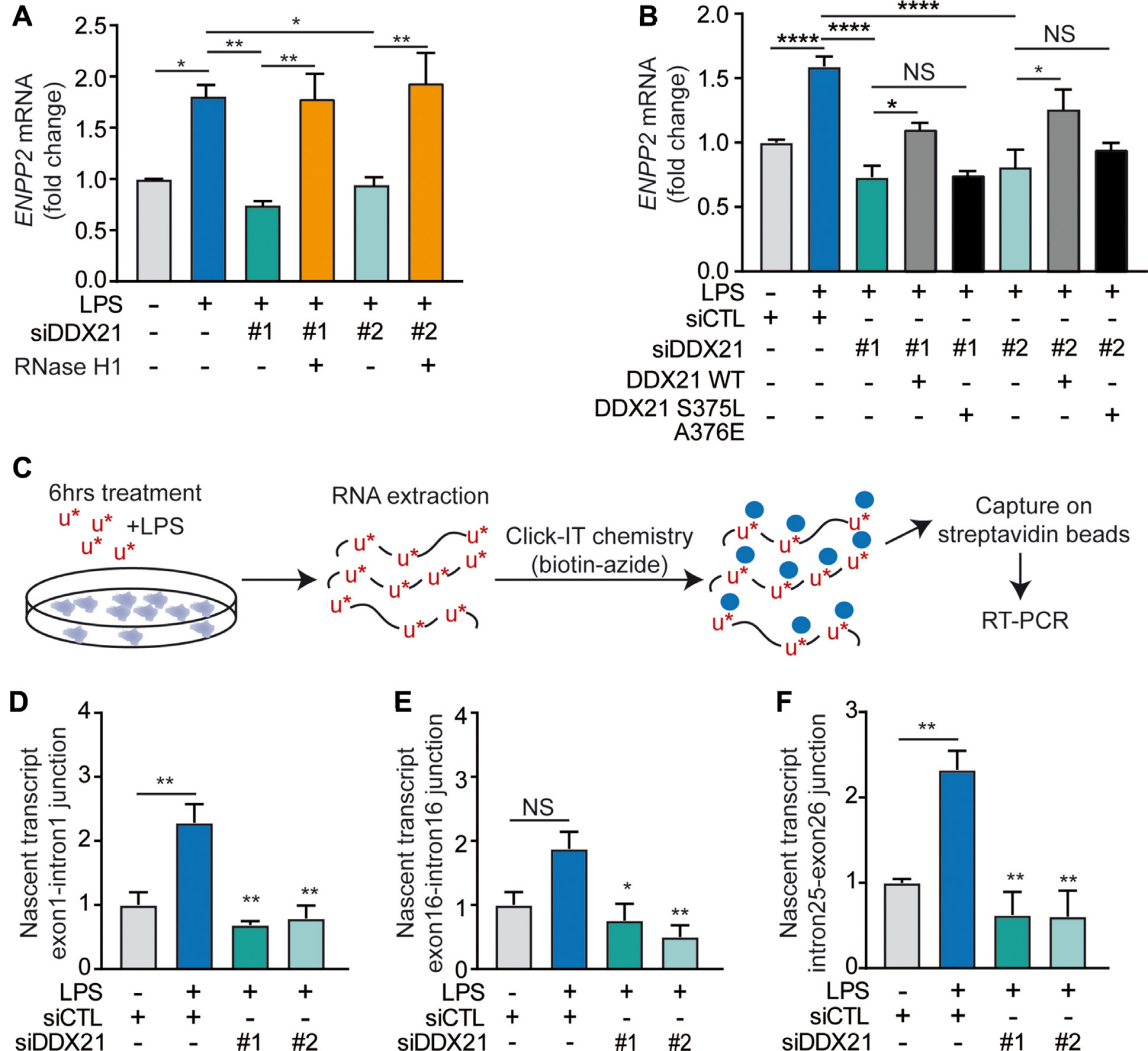
R-loops resolution at *ENPP2* locus promotes the synthesis of nascent transcript. (**A** and **B**) Endogenous *ENPP2* expression after transfection of siDDX21 and rescue with a vector encoding (A) RNaseH1 or in (B) DDX21 WT or a DDX21 S375L/A376E (catalytic dead mutant), qPCR analyses. (**C**) Schematic representation of the experimental protocol for transcriptional run-on assay using the Click-it technology. HEK 293T cells were pulse labeled with 5-ethynyl uridine (EU) for 6 h, and the EU-incorporated RNAs were biotinylated and captured with streptavidin conjugated beads, followed by RT-PCR. (**D**–**F**) Transcriptional run-on assay indicates that depletion of DDX21 alters *ENPP2* nascent transcript formation. LPS 100 ng/ml, EU 0.2 mM.

During the transcription process, R-loops are naturally formed and constitute a regulatory system. Although, previous studies revealed that R-loops have beneficial functions in gene expression (23), growing evidence suggest that R-loops detected at exon 2 slowed Pol II progression by creating a chromatin environment favorable for Pol II pausing (40). To investigate the functional role of R-loop in *ENPP2* expression we depleted DDX21 with two non-overlapping siRNAs (Supplementary Figures S8A–C). In some conditions, a plasmid coding for RNaseH1 was also transfected. In these experiments, RNaseH1 completely rescued the expression of *ENPP2* upon LPS treatment, indicating that LPS-induced *ENPP2* expression relies on the resolution of R-loops (Figure 5A). Moreover, to investigate whether the helicase activity of DDX21 is involved in the regulation of *ENPP2* via R-loops resolution, we performed a DDX21 rescue experiment with a helicase-dead mutant DDX21 (S375L/A376E) or with a vector encoding for the wild-type DDX21. Following the knockdown of DDX21 with two non-overlapping siRNAs, we found in rescue experiments that the helicase activity of DDX21 was required to partly restore the expression of *ENPP2* (Figure 5B). In these experiments, we confirmed that DDX21 mutant was similarly expressed compared to wild-type plasmids constructs in cells treated with siRNAs (Supplementary Figure S8D–F).

Next, we performed a transcription run-on experiment to assess the impact of siRNA-mediated depletion of DDX21 on the level of *ENPP2* nascent transcript. To capture nascent RNAs, HEK 293T cells were pulse labeled with 5-ethynyl uridine (EU) for 6 h and the EU-incorporated RNAs were biotinylated and purified with streptavidin conjugated beads (Figure 5C). Interestingly, we found that the knockdown of DDX21 impaired the production of nascent *ENPP2* transcripts suggesting that DDX21 promotes the elongation of transcription at *ENPP2* locus (Figure 5D–F).

### A distant-acting enhancer contacts the promoter region of *ENPP2* and controls its expression

**Figure 6.**
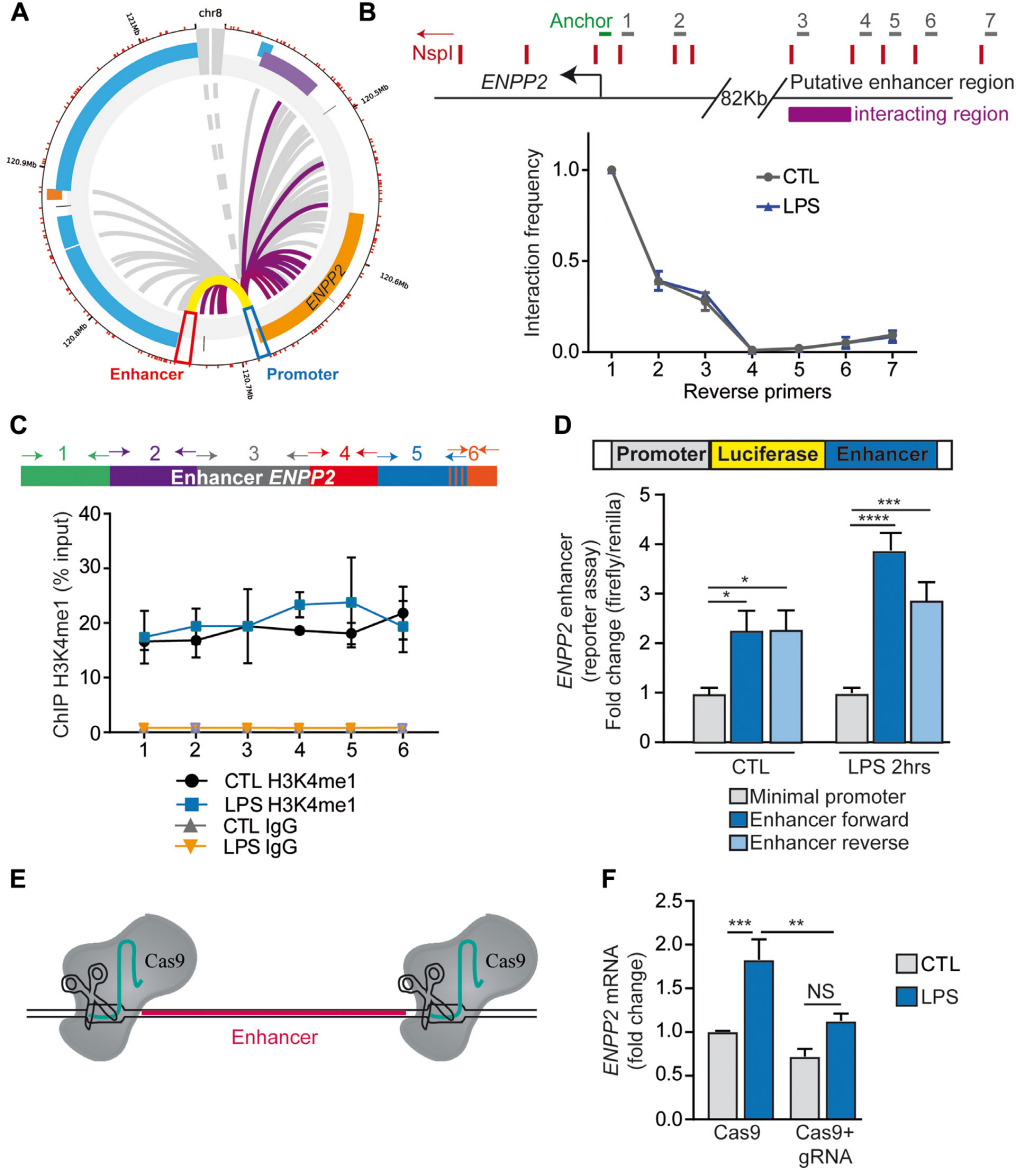
A distant-acting enhancer contacts the promoter region of *ENPP2* and controls its expression. (**A**) Circos plot showing interactions between *ENPP2* TSS and a putative enhancer located at 82 kb, dataset from CD34+ cells. (**B**) 3C experiments replicating this interaction in HEK 293T cells. LPS treatment did not modulate this interaction. (**C**) H3K4me1 enrichment at the enhancer region, as measured by ChIP-qPCR, using six sets of primers covering the putative enhancer region. (**D**) Luciferase reporter assay demonstrating that the region has enhancer activity, independently of its orientation. (**E**) Scheme depicting the deletion of the enhancer region by using the CRISPR-Cas9 technology. Cas9 enzyme was transfected simultaneously with two gRNA targeting 5′ end and 3′ end of the enhancer region. (**F**) Endogenous expression of *ENPP2* after CRISPR-Cas9-mediated deletion of the enhancer, as measured by q-RT-PCR. LPS 100 ng/ml.

To evaluate whether *ENPP2* locus has chromatin interactions we used available data obtained from promoter capture Hi-C (PCHi-C) experiments (www.chicp.org) (35). PCHi-C is a web-based tool that allows the detection of physical chromatin interactions between distal DNA regulatory elements and 31 253 annotated promoters in 17 human primary blood cell types. Interrogation of PCHi-C in CD34+ cells revealed a physical interaction of the promoter region of *ENPP2* with a putative regulatory element, which is located at 82 kb from the TSS (Figure 6A). To determine if this physical interaction was conserved in HEK 293T cells we conducted a 3C assay (41). 3C technology employs formaldehyde crosslink to preserve endogenous three-dimensional structures and digestion of genomic DNA using a restriction enzyme (in this case NspI). Samples were ligated under conditions that favor the union of DNA fragments that are physically connected and qPCR was used to determine the frequency of interaction of each restriction fragments with an anchor primer located at the TSS of *ENPP2*. In HEK 293T cells, we found that the TSS of *ENPP2* and fragment #3–4, corresponding to the 5′ portion of the putative regulatory element, were positioned in close proximity on chromatin (Figure 6B). The treatment with LPS did not modify the chromatin looping pattern (Figure 6B). Therefore, these results suggested that a distant regulatory element could function as an enhancer for *ENPP2*.

To verify this hypothesis we performed ChIP-qPCR with an H3K4me1 antibody with six different primer sets to cover this potential regulatory locus. We found that this region was strongly enriched for H3K4me1, which is consistent with a potential enhancer (Figure 6C). In addition, we cloned this sequence in a reporter plasmid containing a minimal promoter and the luciferase gene (Figure 6D). Importantly, this region, both in forward and reverse orientation, increased by 2-fold the luciferase activity before LPS activation (Figure 6D). Upon LPS stimulation, the enhancer effect on luciferase activity was more pronounced (Figure 6D). Together, these results suggest that the putative enhancer region has a transcriptional effect. At last, to investigate whether this enhancer controls the expression of *ENPP2*, genome editing was performed with the clustered regularly interspersed short palindromic repeats (CRISPR)-Cas9 system to specifically delete this enhancer (Figure 6E). CRISPR-Cas9-mediated deletion of the enhancer region decreased the rise of *ENPP2* after LPS stimulation (Figure 6F). In these experiments, the efficiency of enhancer deletion was confirmed by q-RT-PCR using 5 sets of primers spanning the enhancer region and control regions located upstream and downstream of the predicted cut sites (Supplementary Figure S9A). The residual signal in selected cells is likely related to monoallelic deletion (Supplementary Figure S9B). Taken together, these data revealed that a distant-acting enhancer contacts the promoter region of *ENPP2* and exerts a control on gene expression.

### Enhancer-mediated enrichment of JMJD3–DDX21 at *ENPP2*

**Figure 7.**
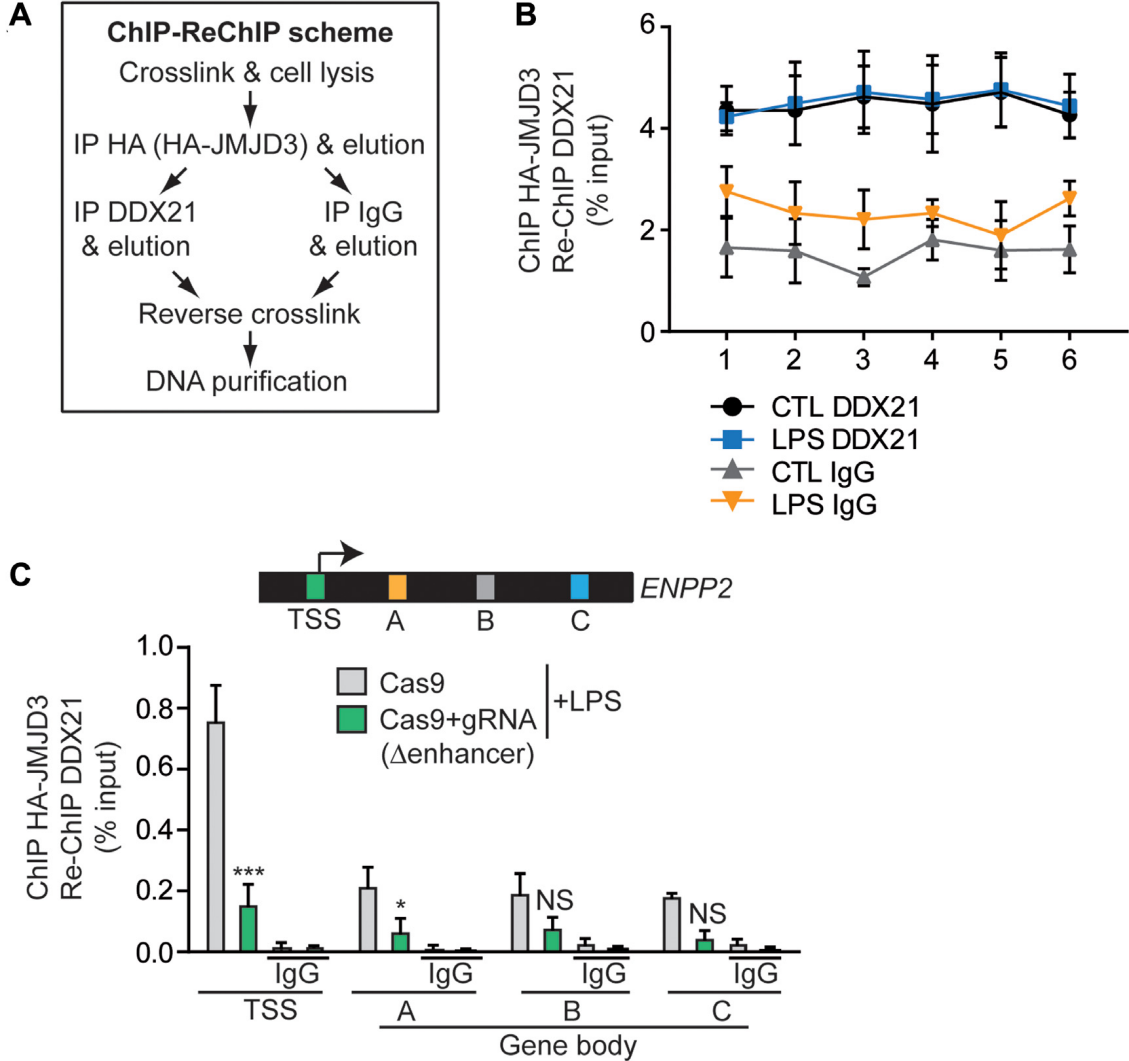
Enhancer-mediated enrichment of JMJD3–DDX21 at *ENPP2*. (**A**) Schematic of the ChIP-reChIP protocol. (**B**) Binding of JMJD3–DDX21 complex on *ENPP2* enhancer region, as measured in ChIP-reChIP experiments and (**C**) after deletion of enhancer region by CRISPR-Cas9 technology in presence of LPS. LPS 100 ng/ml.

The above observations raised the intriguing hypothesis that JMJD3–DDX21 enrichment at the TSS of *ENPP2* could be mediated by DNA looping with the enhancer. Interestingly, in ChIP-re-ChIP experiment, we found that JMJD3–DDX21 complex was present at the enhancer and stimulation with LPS did not modify the signal at this locus (Figure 7A-B). Interestingly, in ChIP re-ChIP experiment, the deletion of the distant-acting enhancer dramatically decreased the co-recruitment of JMJD3 and DDX21 at TSS and through the gene body of *ENPP2* (Figure 7C). Hence, these data indicate that in response to an inflammatory stimulus co-occupancy of JMJD3–DDX21 at the *ENPP2* locus is fostered by the presence of a distant-acting enhancer.

## DISCUSSION

**Figure 8.**
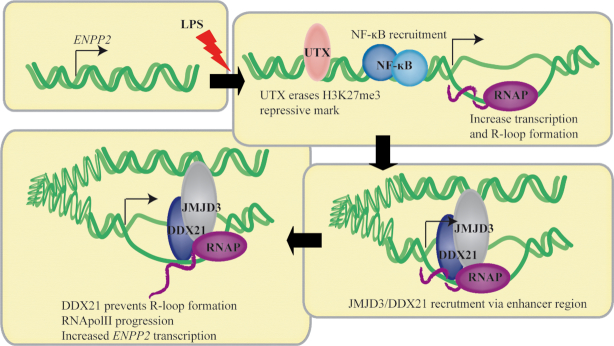
Working model of the role of JMJD3 in the regulation of *ENPP2* transcription. LPS increases transcription at the *ENPP2* locus through a NF-кB pathway coinciding with a reduction of the H3K27me3 histone mark, which is promoted by the recruitment of UTX. Concomitantly, enhancer-mediated enrichment of JMJD3–DDX21 complex at the TSS stimulates transcription elongation through R-loops resolution.

*ENPP2* is overexpressed during inflammatory state and plays a significant role in diverse pathological conditions. In this work, we show that *ENPP2* is induced in response to LPS via κB response elements in the promoter. UTX and not JMJD3 promotes the removal of H3K27me3 at *ENPP2* promoter, characterized by bivalent chromatin marks. On the other hand, we found that JMJD3 interacts with a complex formed with RNA Pol II and DDX21, which is enriched at the promoter of *ENPP2* via a distant-acting enhancer. In turn, JMJD3-dependent recruitment of DDX21 to the promoter reduces the formation of R-loops and promotes the transcriptional process at the *ENPP2* locus (Figure 8).

The expression of *ENPP2* is increased in different tumors including hepatocellular carcinoma, melanoma, ovarian and breast cancers. *ENPP2*/ATX-mediated production of LPA promotes cell motility and metastasis. Also, overexpression of *ENPP2* has been documented during arthritis and the production of LPA by *ENPP2*/ATX plays a role in the development of cardiovascular disorders (42,43). Previous work highlighted that tumor necrosis alpha (TNFα) promoted via a NF-κB pathway the expression of *ENPP2* in Hep3B cells (3). However, the molecular process whereby *ENPP2* locus is controlled by NF-κB signaling was still largely undocumented. In the present work, we underlined that two κB sites located at −642/−626 (κB1) and at −333/−317 (κB2) in the promoter region play a crucial role in LPS-mediated expression of *ENPP2*. In reporter assay, the deletion of one κB consensus motif was sufficient to abrogate the response to LPS. These data were consistent with the recruitment of p65 at these κB sites in ChIP assay as well as the increased reporter activity following the transfection of a p65 encoding vector.

Previous work highlighted that JMJD3 plays an important role in controlling the inflammatory response at different loci (44,45). The underlying mechanism by which this enzyme facilitates transcription elongation involves a binding to promoter and gene bodies, thus allowing the release of paused RNA Pol II, and its progression along the gene body. In this work, we found that LPS treatment increased the recruitment of JMJD3 to the TSS and gene body of *ENPP2*, which is in line with previous work (11,17). Moreover, we found that several proteins interact with JMJD3 and are modulated by inflammatory stimuli such as LPS. GO term analysis of the interactome showed an enrichment for RNA processing. One of the top interactome that increased following LPS treatment was DDX21, an RNA helicase. Recent data have shown that DDX21 unwinds R-loops in G quadruplex and promotes the transcriptional process of several ribosomal genes and genes transcribed by RNA pol II (25). In the present work, we underscored that DDX21 was also required for LPS-mediated expression of *ENPP2*. Of note, siRNA-mediated knockdown of DDX21 or JMJD3 increased the formation of R-loops at *ENPP2* transcriptional unit. These data thus highlighted that likely JMJD3 is required to promote the recruitment of DDX21 to the TSS and gene body of *ENPP2*. In this regard, ChIP re-ChIP experiments confirmed that a complex formed by JMJD3 and DDX21 was recruited at the TSS and gene body of *ENPP2* in response to inflammatory stimulus. Interestingly, this molecular mechanism seems conserved in U937 cells, a human monocyte line (Supplementary Figure S10). Firstly, in U937 cells, *ENPP2* mRNA was also significantly increased at 6 h after the addition of 100 ng/ml of LPS to growth the medium (Supplementary Figure S10A). Secondly, Re-ChIP experiments recovered the JMJD3–DDX21 co-recruitment at TSS of *ENPP2*, which is enriched in presence of LPS (Supplementary Figure S10B and C).

Whether the enzymatic activity of JMJD3 is necessary to promote gene expression at bivalent promoters is subject to some controversies, but recent evidence indicates that several loci relying on JMJD3 for the transcription process do not rely on the enzymatic function (11,12,46). These data are in line with experiments showing that JMJD3 is recruited at the gene body of several genes and interacts with RNA Pol II in promoting transcriptional elongation, a function independent from its catalytic activity (17). We found that the promoter of *ENPP2* is bivalent with H3K4me3 and H3K27me3 marks. After LPS treatment, there is a removal of H3K27me3 at κB sites. However, the removal of H3K27me3 did not rely on JMJD3. Consistently, in rescue experiment the transfection of a wild-type or a catalytically inactive mutant JMJD3 vector restored LPS-mediated expression of *ENPP2*. We found that LPS-mediated expression of *ENPP2* was abrogated by the silencing of JMJD3 or UTX. Of interest, we highlighted that UTX was involved in the removal of H3K27me3 mark at *ENPP2* locus. UTX is a H3K27me3 demethylase, which is expressed by the X chromosome. Hence, though this hypothesis requires further evaluation it is possible that higher circulating level of ENPP2/ATX documented in women is explained, at least in part, by the role of UTX, which is known to escape X inactivation (47,48).

Distant acting enhancer regulates gene expression through looping (49). Models suggest that contact-mediated interaction between enhancers and promoters provide an enrichment of the transcriptional machinery, a process whereby transcription is reinforced. Promoter capture HI-C provides high-resolution mapping of chromatin contacts (35). By analyzing publicly available capture Hi-C datasets, we identified a region located at 82 kb from *ENPP2*, which interacted with the promoter. Importantly, using 3C assay we documented that this DNA looping was conserved in HEK 293T cells. In addition, we reported a significant enrichment for H3K4me1, which is consistent with an enhancer. Functional assessment in minimal reporter assay showed that this region increased the luciferase activity by 2-fold before activation with LPS. Basal and LPS-mediated expression of *ENPP2* were significantly decreased in cells in which this region had been deleted by CRISPR-Cas9. Collectively, these data suggested that this region is a distant-acting enhancer, which participates to the regulation of *ENPP2* expression. We found that deleting the enhancer largely abrogated in ChIP re-ChIP the co-occupancy of JMJD3–DDX21 at the *ENPP2* locus. Hence, these data highlighted that interplays at this locus control the expression of *ENPP2* though an enhancer-mediated enrichment of a complex formed by JMJD3–DDX21 at the promoter.

In conclusion, characterization of the *ENPP2* locus showed that inflammation-mediated expression is controlled through several processes including H3K27me3 demethylases UTX and JMJD3. Also, a distant-acting enhancer promotes the enrichment of a complex formed by JMJD3–DDX21 at *ENPP2*, which allows nascent transcript production by removing R-loops. Further work is necessary to identify if similar processes operate at different loci during inflammation.

## DATA AVAILABILITY

David Bioinformatics Ressources 6.8 is an open source available at https://david.ncifcrf.gov/. Promoter capture Hi-C (PCHi-C) is a web-based tool freely available at www.chicp.org without login requirement.

## Supplementary Material

gkz560_Supplemental_Files
