# Supplementary material for: Enhancer-mediated enrichment of interacting JMJD3–DDX21 to ENPP2 locus prevents R-loop formation and promotes transcription
Source: Nucleic Acids Res. 2019 Jun 28;47(16):8424–38. doi: 10.1093/nar/gkz560 (PMC6895255; doi:10.1093/nar/gkz560)
Supplement: gkz560_Supplemental_Files [file gkz560_supplemental_files.zip › SupplementaryFigures_170619.pdf]

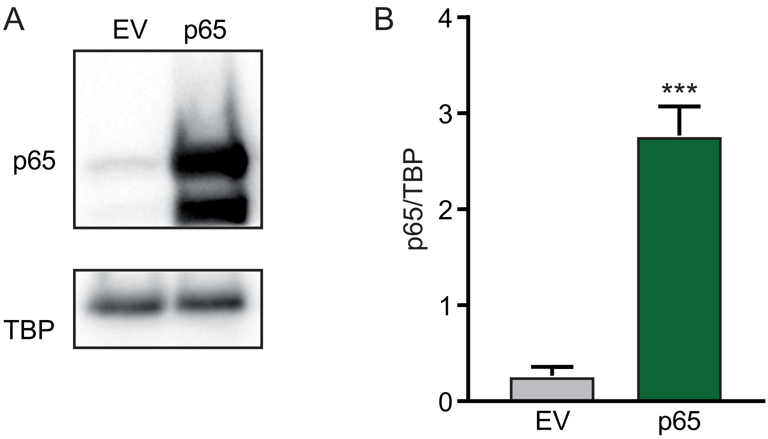

Supplementary figure 1: Level of p65 overexpression in HEK 293T. A) Representative western blot. B) Western blots quantifications.

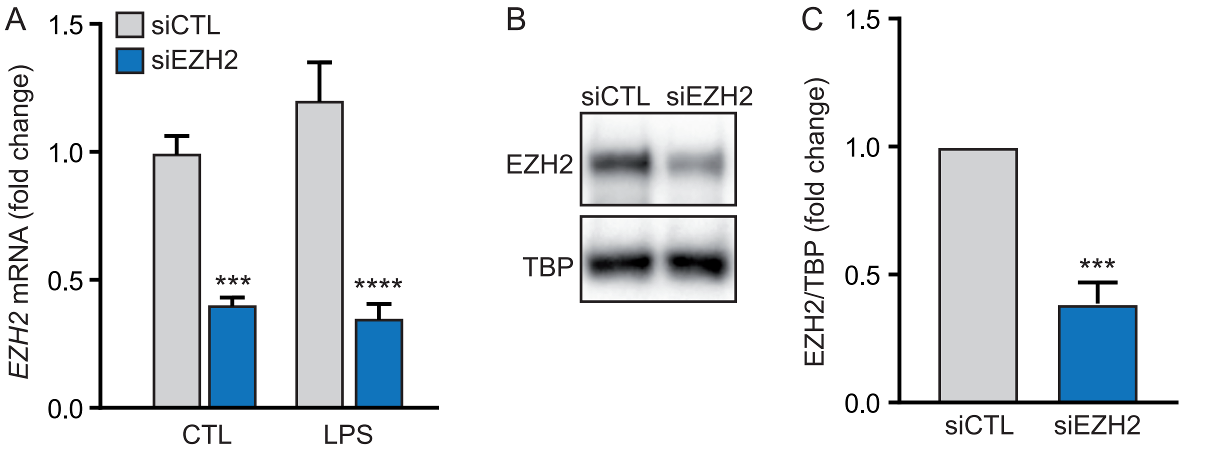

Supplementary figure 2: Efficiency of EZH2 siRNA in HEK 293T cells. A) qPCR analysis. B) Representative western blot. C) Western blots quantifications. LPS 100ng/ml.

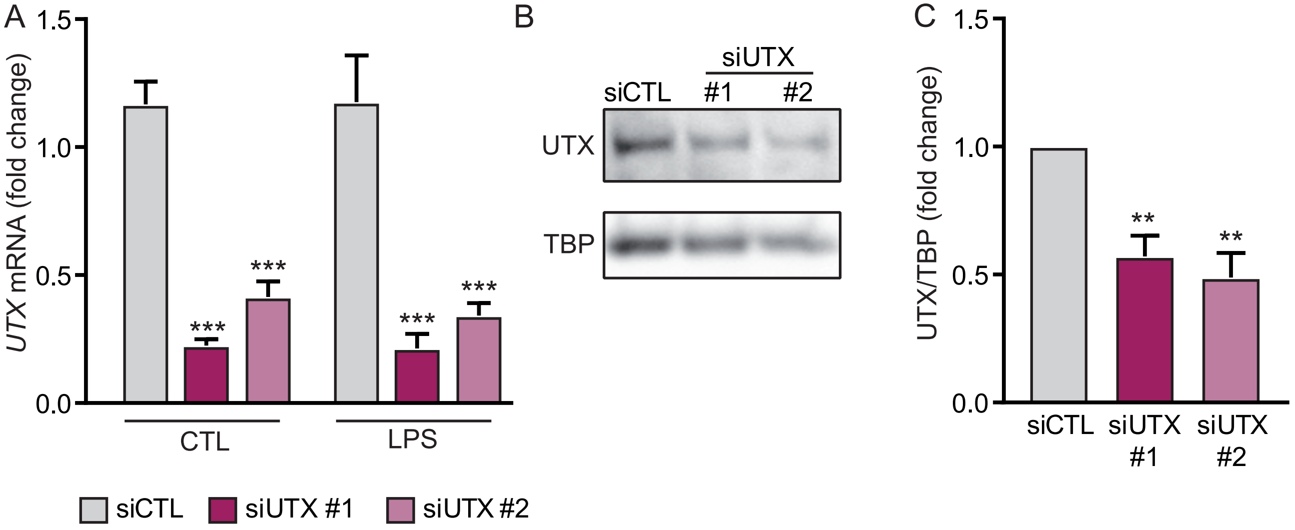

Supplementary figure 3: Efficiency of UTX siRNAs in HEK 293T cells. A) qPCR assays. B) Representative western blot. C) Western blots quantifications. LPS 100ng/ml.

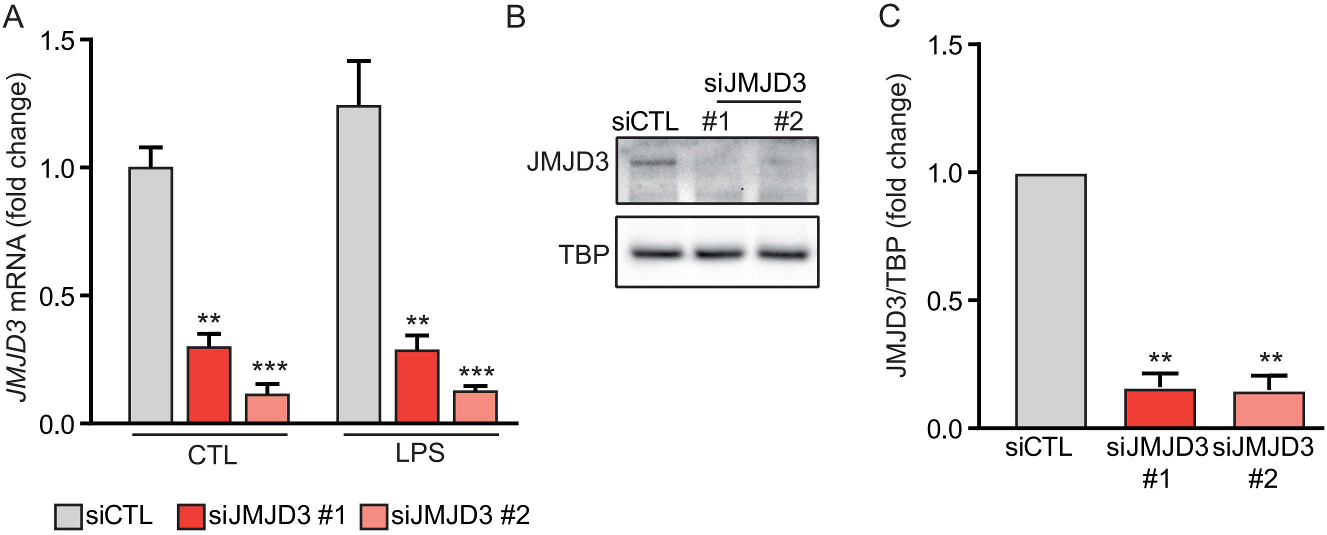

Supplementary figure 4: Efficiency of JMJD3 siRNAs in HEK 293T cells. A) qPCR assays. B) Representative western blot. C) Western blots quantifications. LPS 100ng/ml.

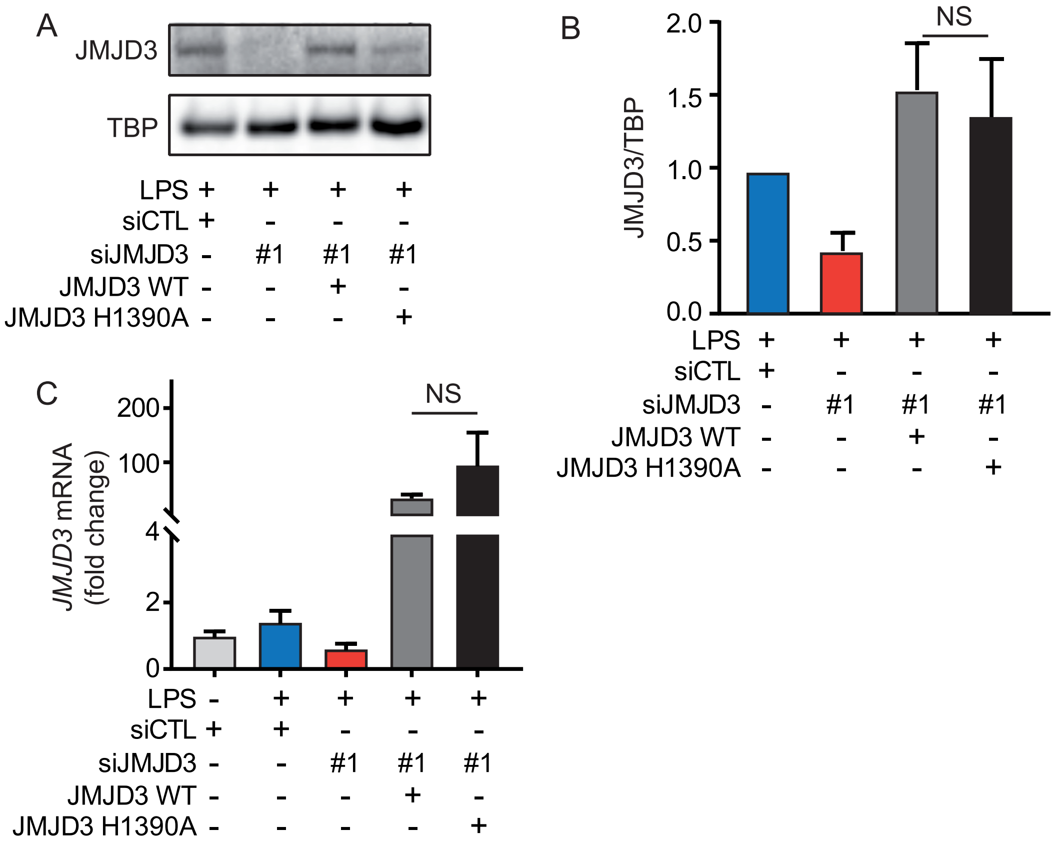

Supplementary figure 5: Overexpression of MYC-JMJD3. (A) Representative western blots using JMJD3 antibodies on HEK 293T transfected cell extracts (B) quantifications. C) qPCR showing *JMJD3* expression level in rescue experiments. LPS 100ng/ml.

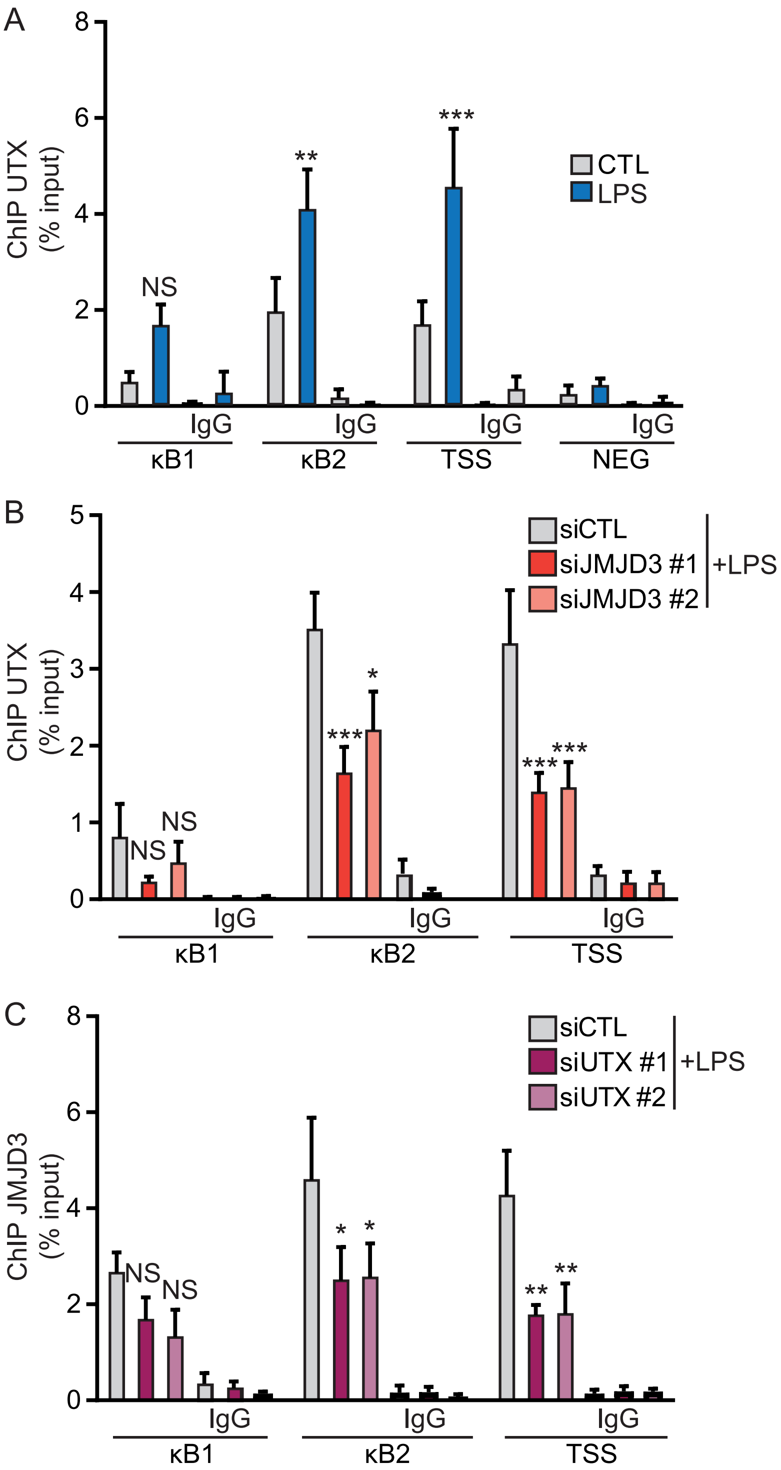

Supplementary figure 6: ChIP-qPCR assays showing crosstalk between UTX and JMJD3. A-B) UTX recruitment at the *ENPP2* promoter, effect of (A) LPS and (B) JMJD3 siRNAs. C) UTX depletion alters JMJD3 recruitment at the *ENPP2* promoter. LPS 100ng/ml.

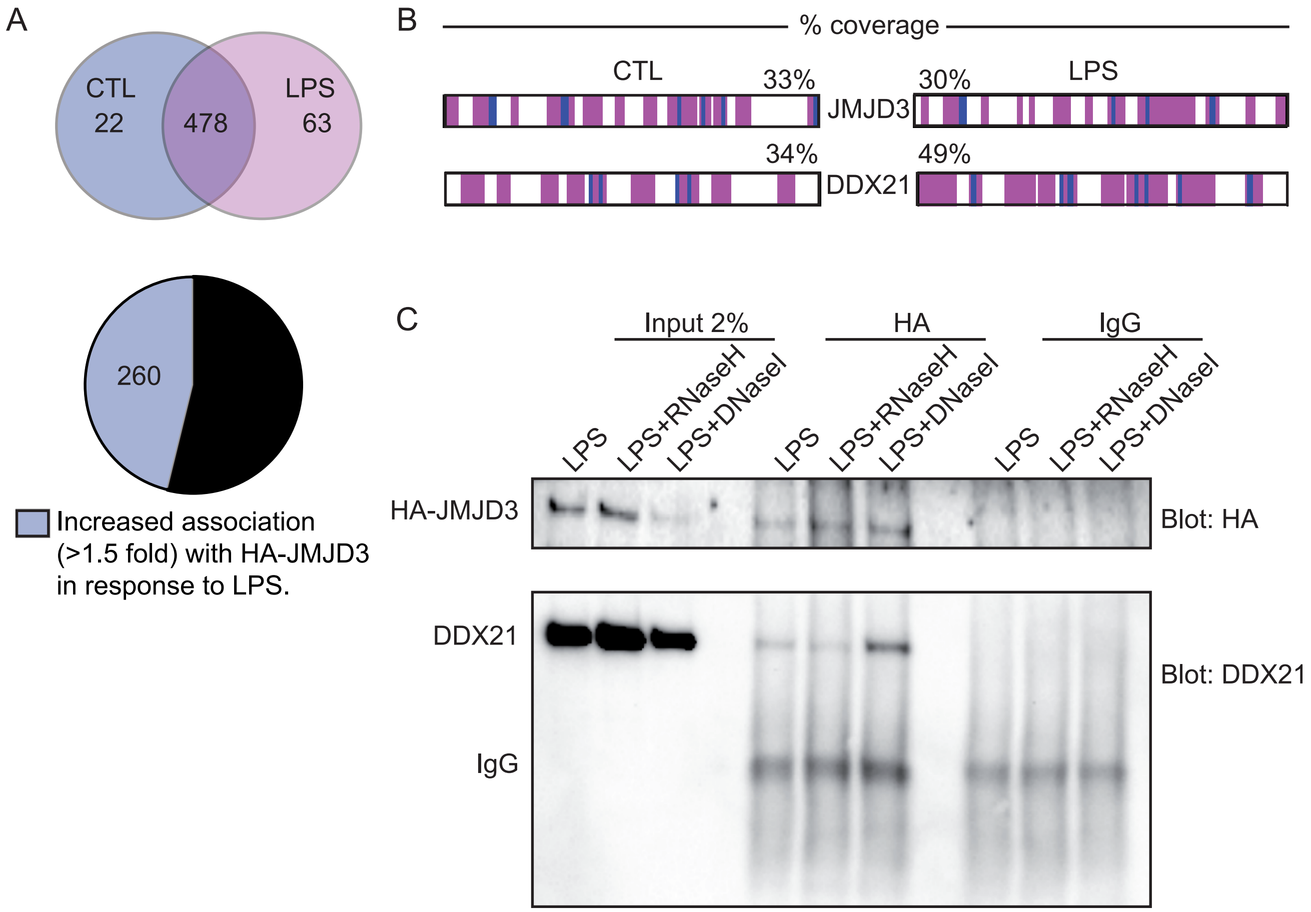

Supplementary figure 7: JMJD3 and DDX21 interaction. A) Venn diagram of mass spectrometry results; from total proteins, 260 showed increased association with HA-JMJD3 with a fold change superior to 1.5. B) JMJD3 and DDX21 coverage in mass spectrometry analysis. C) Co-immunoprecipitation of HA-JMJD3 and endogenous DDX21. LPS100ng/ml, RNase H 40U/ml, DNase I 25U/ml.

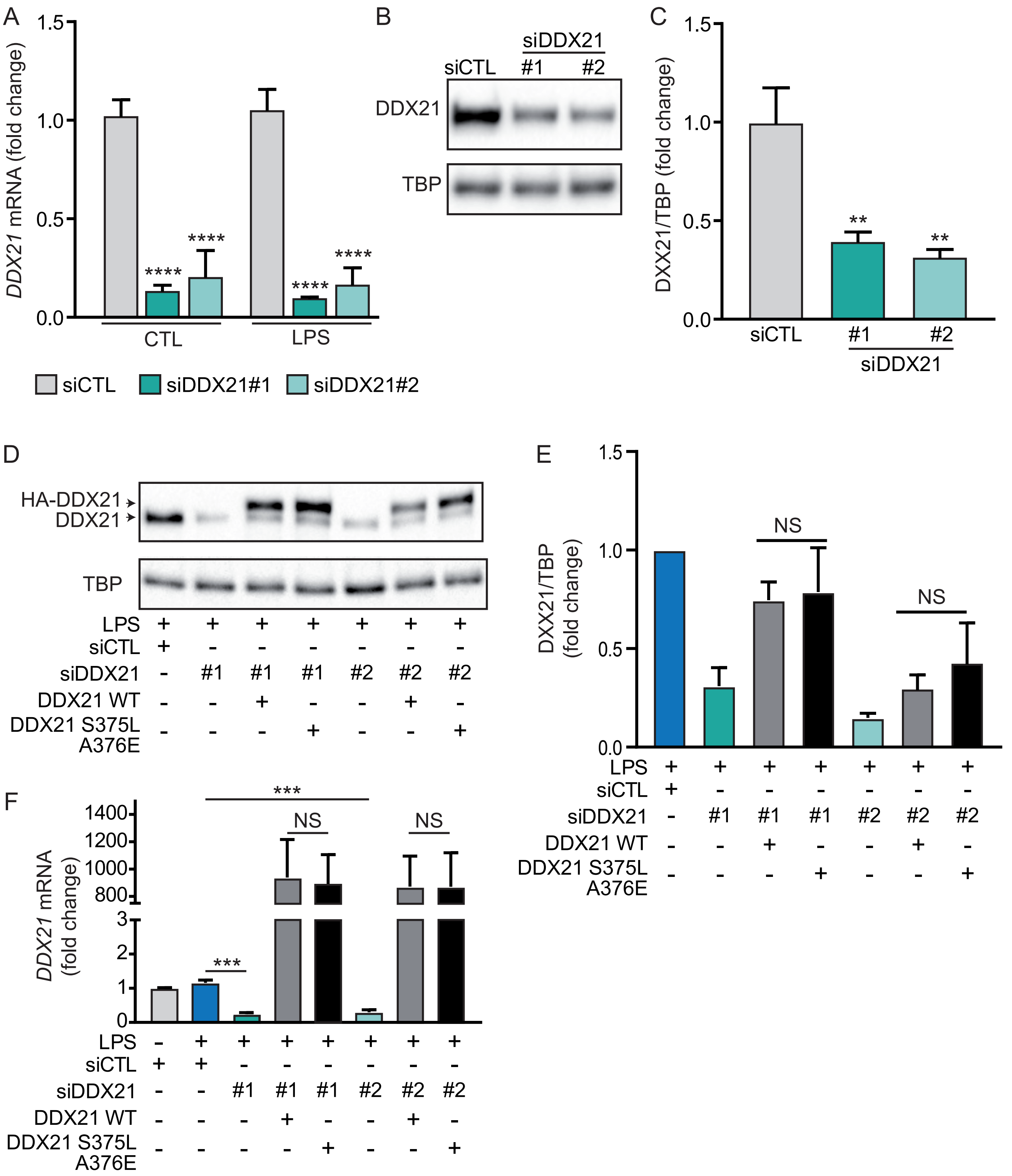

Supplementary figure 8: DDX21 overexpression in HEK-293T cells. A-C) DDX21 siRNAs efficiency (A) qPCR analysis (B) representative western blot (C) quantifications. D-F) Overexpression of DDX21 in HEK-293T cells during rescue experiments (D) representative western blot (E) protein quantifications and (F) qPCR analysis. LPS 100ng/ml.

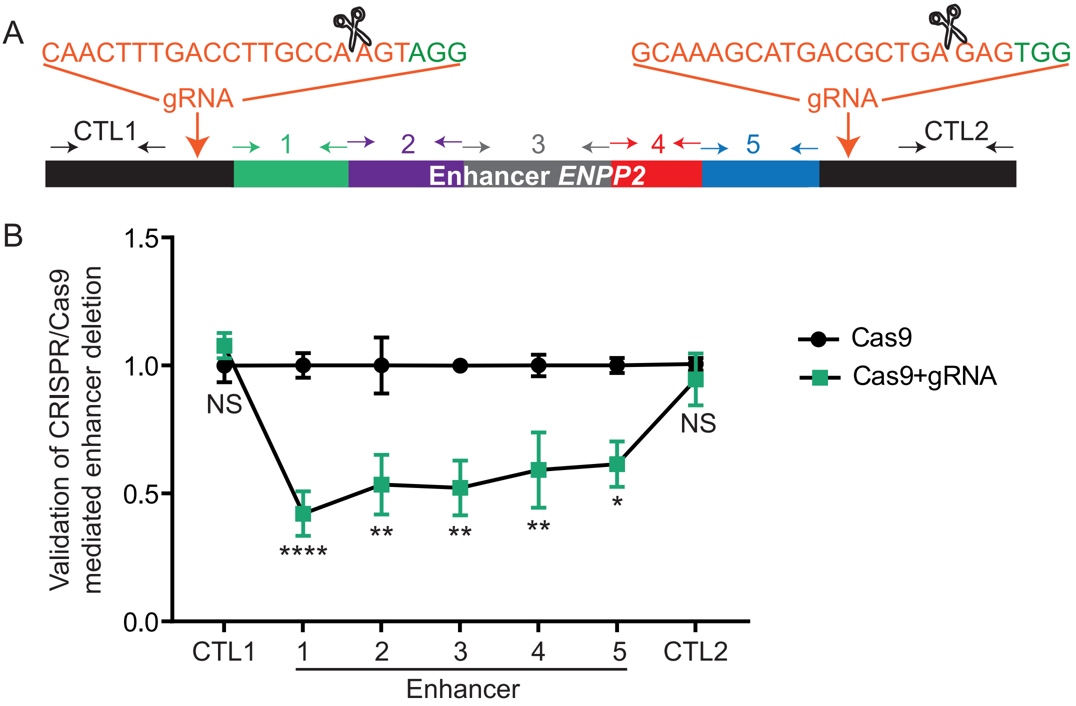

Supplementary figure 9: CRISPR/Cas9-mediated enhancer deletion in HEK 293T cells. (A) Scheme of deletion region with gRNA sequences (PAM sequences are shown in green) and sites of qPCR primers. B)The efficiency of enhancer deletion was confirmed by multiple qPCR analyses.

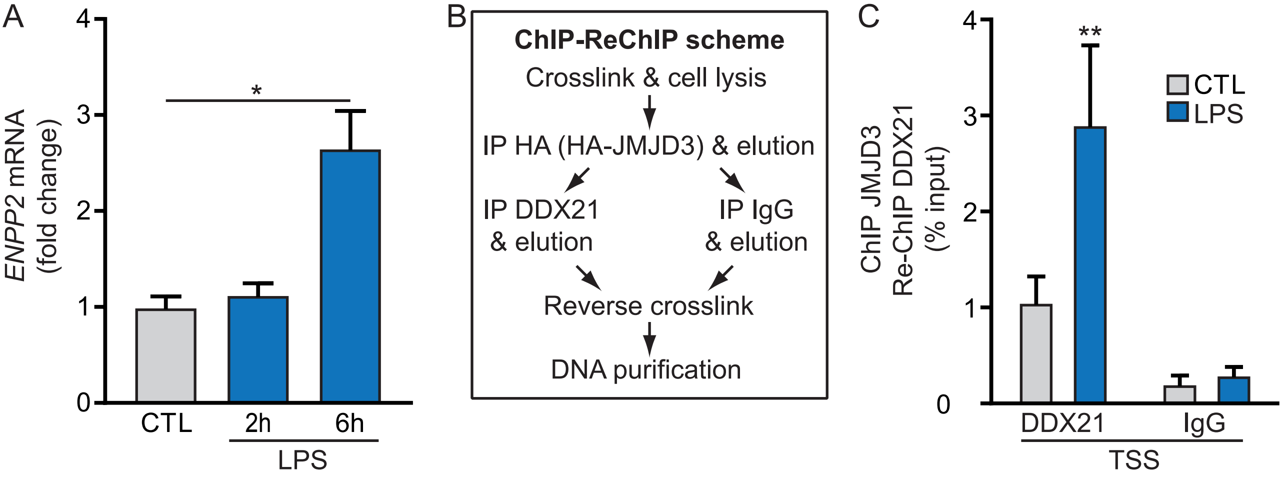

Supplementary figure 10: *ENPP2* regulation in U937 cells. A) qPCR analysis of *ENPP2* expression level in response to LPS treatment. B) JMJD3 and DDX21 recruitment at *ENPP2* TSS, shown by ChIP re-ChIP experiment, at basal level and in response to LPS treatment. LPS 100ng/ml.
